# An artificially simulated outbreak of a respiratory infectious disease

**DOI:** 10.1186/s12889-020-8243-6

**Published:** 2020-01-30

**Authors:** Zuiyuan Guo, Shuang Xu, Libo Tong, Botao Dai, Yuandong Liu, Dan Xiao

**Affiliations:** 1 Department of Disease Control, Center for Disease Control and Prevention in Northern Theater Command, No. 6, Longshan Road, Shenyang, 110034 China; 2 Liaoning Agricultural Development Service Center, Shenyang, China; 3 China National Clinical Research Center for Neurological Diseases, Beijing Tian Tan Hospital, No. 119, South 4th Ring Road West, Fengtai District, Beijing, China

**Keywords:** Respiratory infectious diseases, Adenovirus type 7, Outbreak, Model, Prevention, Control

## Abstract

**Background:**

Outbreaks of respiratory infectious diseases often occur in crowded places. To understand the pattern of spread of an outbreak of a respiratory infectious disease and provide a theoretical basis for targeted implementation of scientific prevention and control, we attempted to establish a stochastic model to simulate an outbreak of a respiratory infectious disease at a military camp. This model fits the general pattern of disease transmission and further enriches theories on the transmission dynamics of infectious diseases.

**Methods:**

We established an enclosed system of 500 people exposed to adenovirus type 7 (ADV 7) in a military camp. During the infection period, the patients transmitted the virus randomly to susceptible people. The spread of the epidemic under militarized management mode was simulated using a computer model named “the random collision model”, and the effects of factors such as the basic reproductive number (*R*_0_), time of isolation of the patients (TOI), interval between onset and isolation (IOI), and immunization rates (IR) on the developmental trend of the epidemic were quantitatively analysed.

**Results:**

Once the *R*_0_ exceeded 1.5, the median attack rate increased sharply; when *R*_0_ = 3, with a delay in the TOI, the attack rate increased gradually and eventually remained stable. When the IOI exceeded 2.3 days, the median attack rate also increased dramatically. When the IR exceeded 0.5, the median attack rate approached zero. The median generation time was 8.26 days, (95% confidence interval [CI]: 7.84–8.69 days). The partial rank correlation coefficients between the attack rate of the epidemic and *R*_0_, TOI, IOI, and IR were 0.61, 0.17, 0.45, and − 0.27, respectively.

**Conclusions:**

The random collision model not only simulates how an epidemic spreads with superior precision but also allows greater flexibility in setting the activities of the exposure population and different types of infectious diseases, which is conducive to furthering exploration of the epidemiological characteristics of epidemic outbreaks.

## Background

Respiratory infectious diseases, especially strains of influenza A and ADV such as H1N1, H7N9, ADV 7, and ADV 55, often lead to worldwide outbreaks and seriously endanger human health. For example, from late April to the end of 2009, the local H1N1 flu epidemic peaked in most countries, and approximately 70,000 laboratory-confirmed hospitalized patients and 2500 fatal cases were observed across 19 countries or administrative regions [1, 2]. Epidemics of ADV infection often occur in healthy children or adults in closed or crowded settings (particularly in communities, military recruit training centres, hospitals, and chronic care facilities) worldwide [3–6]. Fatality rates for untreated severe adenovirus-associated pneumonia or disseminated disease may exceed 50% [7].

ADV 7 outbreaks are very common among military trainees in many countries [8–13], most likely due to the close living quarters of trainees, the persistence of adenoviruses in the environment when infectious agents from epidemic areas enter the camp, the susceptibility of the general population to some variants [14], and low vaccine coverage [15]. These diseases can spread to create a large-scale outbreak in a very short period of time. Some viral strains can cause serious intrapulmonary infection and even lead to death. Therefore, determining the precise timing for disease control and adopting comprehensive scientific measures to control the spread of an epidemic are demanding challenges facing the public health systems of every country. To achieve the objectives discussed above, theoretical research on the dynamics of epidemic transmission is needed, and the time of control and the impact of measures on the attack rate must be quantitatively analysed.

Mathematical models of infectious diseases can help deepen our understanding of the epidemiological distribution of infectious diseases. Currently, the most commonly used model is the Susceptible-Exposed-Infectious-Recovered (SEIR) model, from which many models have been derived and widely adopted to analyse infectious outbreaks of Ebola, tuberculosis, and influenza, among other diseases [16–18]. Indeed, the SEIR model has proven to be critical for revealing the epidemiological characteristics of infectious diseases. However, this model has some limitations in the analysis of outbreaks of respiratory infectious diseases. For example, SEIR-based models frequently assume that the effective contact rate (the number of people infected by one infector within the time unit when all exposed persons are susceptible) is a constant or a continuous function [16–18], i.e., that infectors transmit the virus continuously. In reality, these contacts occur randomly, and time intervals exist between infection events. Furthermore, according to the SEIR model, as long as someone within the population is infected and the effective contact rate is greater than 0, an outbreak will be triggered, and the disease will spread continuously. However, again, in reality, even if someone in the population becomes infected, an epidemic outbreak may not occur, and even without human intervention, outbreaks typically end before all susceptible people become infected. For example, Justin L reported that 35% of students had an influenza-like illness during an H1N1 influenza outbreak in a middle school [19]. Additionally, the SEIR model assumes that all infectors display the same epidemiological characteristics in their effective contact rates, incubation periods, symptom duration, and treatment duration, but these factors vary across patients. For example, Justin L reported that the 95% confidence interval was between 1.0 and 1.8 for the median incubation period for confirmed H1N1 influenza and between 1.7 and 2.6 for the development of symptoms [19]. Another factor to consider is that the activities of the exposure population are not constant. For example, soldiers in military camps train together during the day, and at night, they rest in the dormitory with their squad unit. Thus, the close contacts of the infectors change over time, but the SEIR model fails to reflect this element.

To overcome the limitations of the SEIR model, we sought to establish an individual-level stochastic research model to simulate the spread dynamics of epidemic outbreaks in the real environment. Such models have been used in teaching and research related to the epidemiology of infectious diseases. For example, Eichner M used stochastic computer simulations to examine whether case isolation, contact tracing, and surveillance can extinguish smallpox outbreaks in highly susceptible populations within less than half a year without causing more than 550 secondary cases per 100 index cases [20]. Salathe M modelled the spread of an infection in a “small-world” network based on computer simulations to assess how a personal opinion about vaccination affects the probability of disease outbreak. The study found that the inclusion of opinion formation led to frequent outbreaks in a homogeneously vaccinated population with vaccination coverage of less than 70% [21]. Williams A employed a discrete time simulation environment to model a virtual town that experienced a bioterrorist attack of pneumonic plague and assessed the attack rate under the influence of a mass treatment centre and home isolation. They found that an attack rate of 93% was approximately equal to the expected theoretical attack rate if *R*_0_ = 2.85 [22]. In addition, Cremin I presented a teaching exercise in which an infectious disease outbreak was simulated over a five-day period and found substantial variation in the cumulative attack rate, with between 26 and 83% of the students uninfected at the end of each outbreak [23]. Although these studies employed the concept of individual-level and random contact among people, they did not fully account for certain factors, such as differences in patient contact behaviour during day and night, the time of isolation, and the duration from onset to isolation, which influences morbidity. Therefore, a stochastic model for the prevention and control of outbreaks of respiratory infectious diseases in a military camp is still lacking.

We chose to use ADV 7, which has high incidence and poses serious health threats in the army, to establish a random collision model that simulates the complete occurrence and development of an ADV 7 outbreak with effective intervention measures. This model not only provided greater flexibility in setting the scope of the population’s activities and enabled the depiction of the transmission network of the outbreak but also permitted quantitative analysis of the impact of intervention measures, thereby providing a scientific basis for targeted prevention and control of the outbreak.

## Methods

### Data sources

First, to construct the model, we needed to acquire the necessary parameters, which were derived from a real outbreak in a boot camp. In November 2018, we conducted an epidemiological investigation and analysed prevention and control management of an ADV 7 outbreak in northeastern China. Northern Theater CDC is responsible for investigating and controlling public health emergencies.

The probability distributions and the parameters of the incubation period (the interval from infection to onset of disease), the generation period (the interval between successive onsets of symptoms in a chain of transmission), the symptom duration (the duration of a patient’s clinical symptoms), and the isolation treatment duration (the duration of isolation treatment for a patient) were calculated (Table 1). These parameters were applied directly to the model, and the method of calculation and all data generated or analysed during this study are provided in the Additional file 1.

**Table 1:** Parameters related to the outbreak characteristics of ADV 7

Types	Probability distribution	Parameters
Incubation period	Log-normal distribution	*mean* = 5.25, *sd* = 0.94
Generation period	Weibull distribution	*mean* = 7.36, *sd* = 2.47
Symptom duration	Log-logistic distribution	*mean* = 6.88, *sd* = 1.83
Isolation treatment duration	Log-logistic distribution	*mean* = 11.01, *sd* = 2.50
Basic reproductive number	Normal distribution	*mean* = 5.09, *sd* = 0.26

### Model establishment

**Fig. 1:**
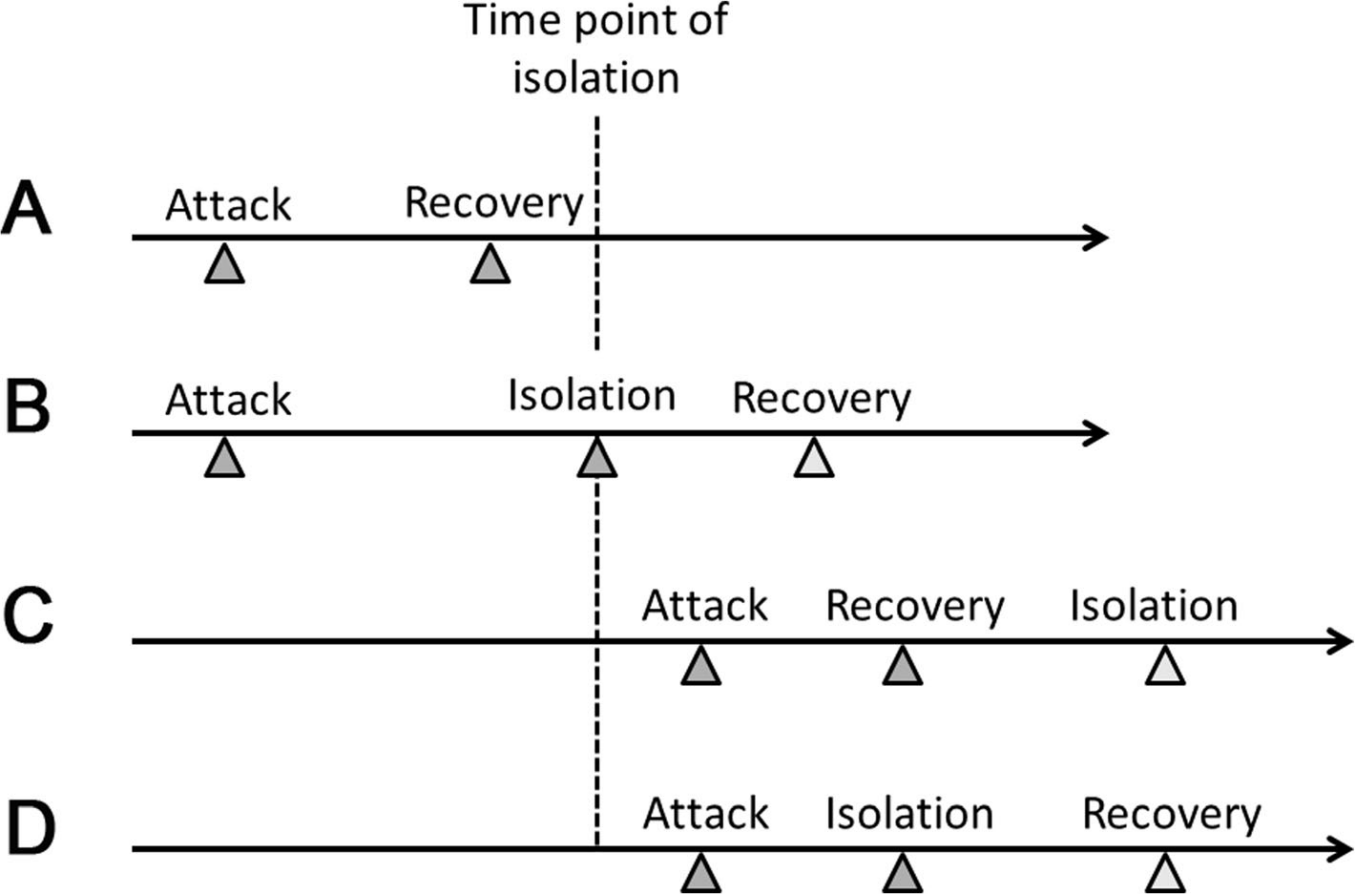
Schematic diagram of the relationship among the time of attack, time of recovery, and TOI. Legend: **a** When a patient’s time of attack is earlier than the TOI, if he recovers before isolation, he will not be isolated. **b** When a patient’s time of attack is earlier than the TOI, if he does not recover before isolation is instated, he will be isolated at the TOI. **c** When a patient’s time of attack is later than the TOI but he recovers before isolation is instated, he will not be isolated. **d** When a patient’s time of attack is later than the TOI, if isolation is instated before the patient recovers, he will be isolated

Second, we established the model according to the following disposal method of respiratory infectious disease outbreaks in Chinese military camps. At the early stage of an outbreak, patients are often treated for the common cold at a clinic in the camp and are still in normal contact with other exposed individuals during treatment, which delays the opportunity for timely isolation and control of the epidemic. When the outbreak reaches a certain level, the CDC will participate in disease control. Patients whose symptoms appeared before the TOI but are still exhibiting symptoms are quickly sent to the hospital. The body temperatures of the exposed individuals are monitored several times daily, and those who develop fevers are quickly sent to the hospital. Patients return to the camp once they recover. The relationships among the time of attack, time of recovery, and TOI of the patients are shown in Fig. 1.

Based on the above description, two enclosed and interconnected systems were simulated in the outbreaks. The first was the military camp where disease transmission originated. Each patient in the model is considered a mass point that walks randomly during the infection period. When patients have contact with other susceptible people, these people become infected. After an incubation period, the infected individuals become patients and transform into new mass points. The collisions continue until no new mass points are generated. Patients are considered infectious only when clinical symptoms begin to appear, and thus the infection period is equivalent to the symptom duration. Each patient transmits the virus at a certain rate through close contact with susceptible people, and the time intervals between infected individuals successively infected by one patient are independent of each other and exponentially distributed.

The second system was the hospital where the isolation treatments were performed. Since medical personnel at the hospital take strict protective measures to prevent nosocomial infections, patients should not transmit the disease to other susceptible people during the treatment duration. Once patients have recovered, they are sent back to the camp to continue their activities with other exposed people. Since recovered individuals have already produced specific antibodies, they will not become infected again.

### Disease transmission network

In addition, we analysed the transmission network of ADV 7 and mapped it using Gephi 0.9.2 under the assumption that the entire exposed population was susceptible and that patients could transmit viruses to susceptible individuals without isolation treatment.

### Factors affecting the outbreak

The model we envisaged was simulated on a computer and was based on a description of the epidemic. In our model, we set up a total of 50 squad units with 10 people in each unit. Military drills are from 06:00 to 18:00 h daily; during these times, all subjects gather to participate in training or learning, and the virus is freely spread among the crowd. By contrast, during non-military drill periods, the subjects rest in the dormitory with their squad unit, and the virus will spread only within the dormitory. Since all soldiers are male, are approximately 20 years old, and meet a unified standard of physical fitness, we considered the population characteristics to be homogeneous. The effective contact rate, incubation period, symptom duration, and treatment duration for each patient were randomly sampled according to the probability distributions presented in Table 1. In addition, immunization of the exposed population can have an impact on development of the outbreak. We preestablished that a portion of the exposed population gained immunity to the virus through vaccination and that these individuals are randomly distributed among the population.

### Generation period

The generation period was also calculated. According to the literature, the generation period is consistent with a Weibull distribution [19]. Therefore, we estimated this index directly on the basis of the probabilistic characteristic using a bootstrap method (1000 iterations of random sampling). The specific calculation methods are described in the Additional file 1.

### Sensitivity analyses

Finally, we performed sensitivity analyses of four significant parameters to assess the impact on the attack rate. Partial rank correlation coefficients (PRCCs) and Latin hypercube sampling (LHS) were used to conduct sensitivity analyses. PRCC-LHS is an efficient and reliable sampling-based sensitivity analysis method that provides a measure of monotonicity between a set of parameters and the model output after removal of the linear effects of all parameters except the parameter of interest [24, 25]. Each parameter interval (from 0.5 to 1.5 times the average value of the parameters) was divided into *N* smaller and equal intervals, and one sample was selected randomly from each interval [24, 25]. A standard coefficient denoting the correlation between the parameter and the model output was calculated. All analyses were conducted using MATLAB R2019a software (MathWorks, USA, 2019).

## Results

### Disease transmission network

**Fig. 2:**
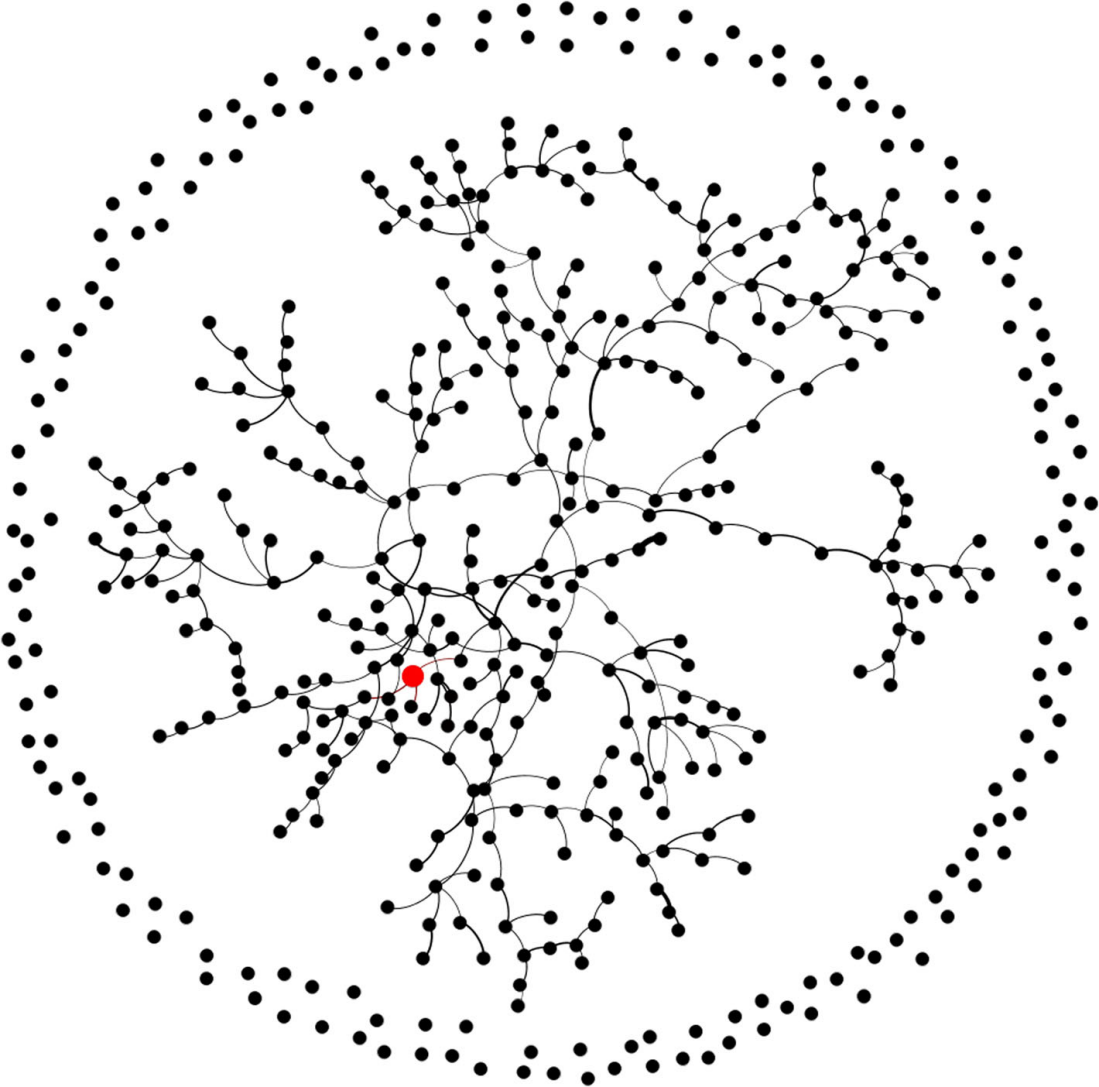
The transmission network of the epidemic outbreak. Legend: Black dots indicate exposed individuals; the red dot indicates the first infector; and lines represent connections between the infector-infected pairs

The transmission network is shown in Fig. 2. The black dots with connecting lines represent patients with infectious connections (as either an infector or an infected individual), totalling 328 people; the dispersed dots around the edge of the graph represent individuals who were exposed but uninfected. The first patient is marked in red; he infected a total of three susceptible people during the infection period.

### Factors affecting the outbreak

**Fig. 3:**
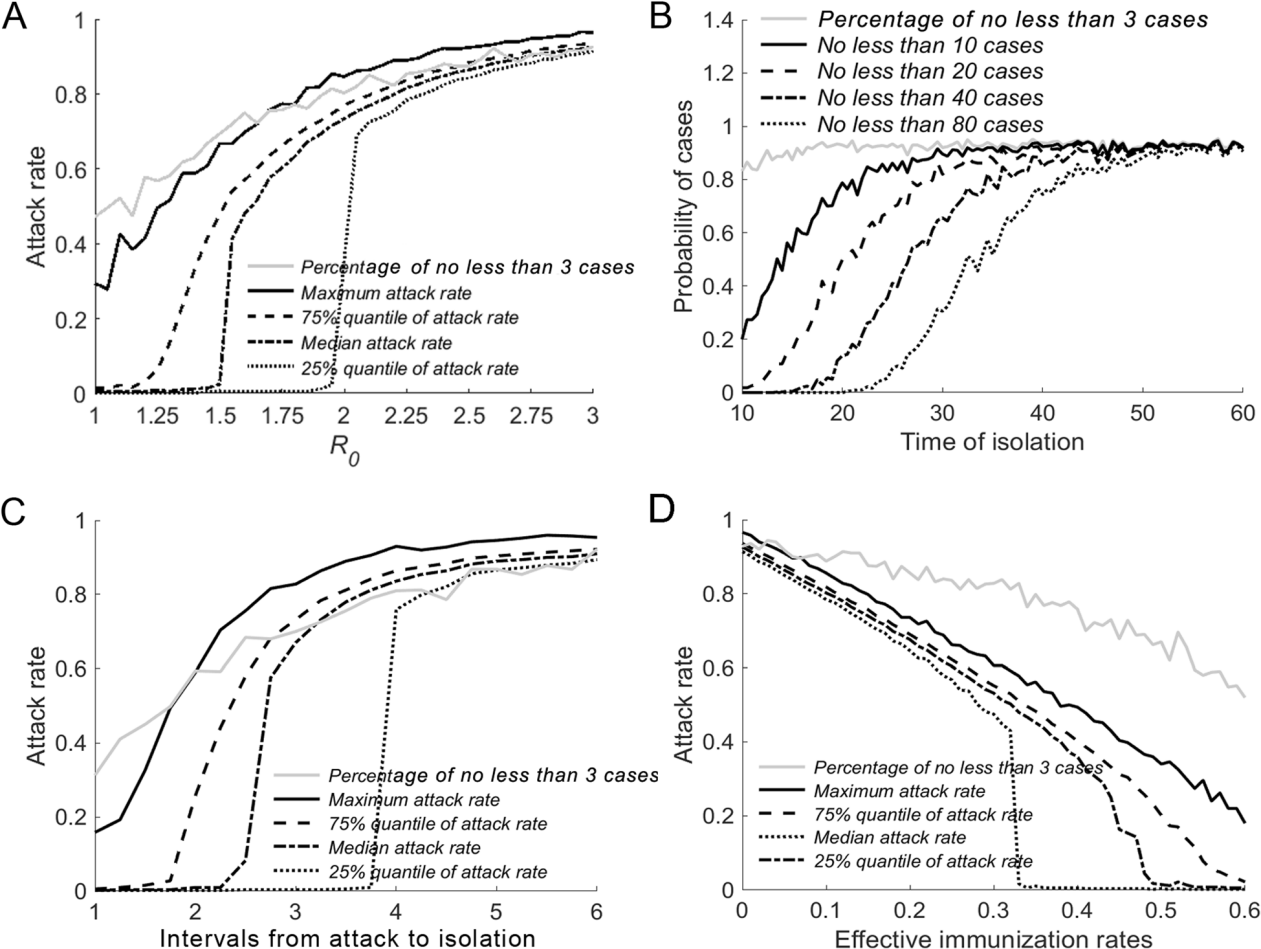
The impact of the four factors on the outbreak. Legend: **a** The impact of *R*_0_ on the attack rate. **b** The effect of TOI on the number of patients. **c** The effect of IOI on the attack rate. **d** The impact of IR on the attack rate

**Fig. 4:**
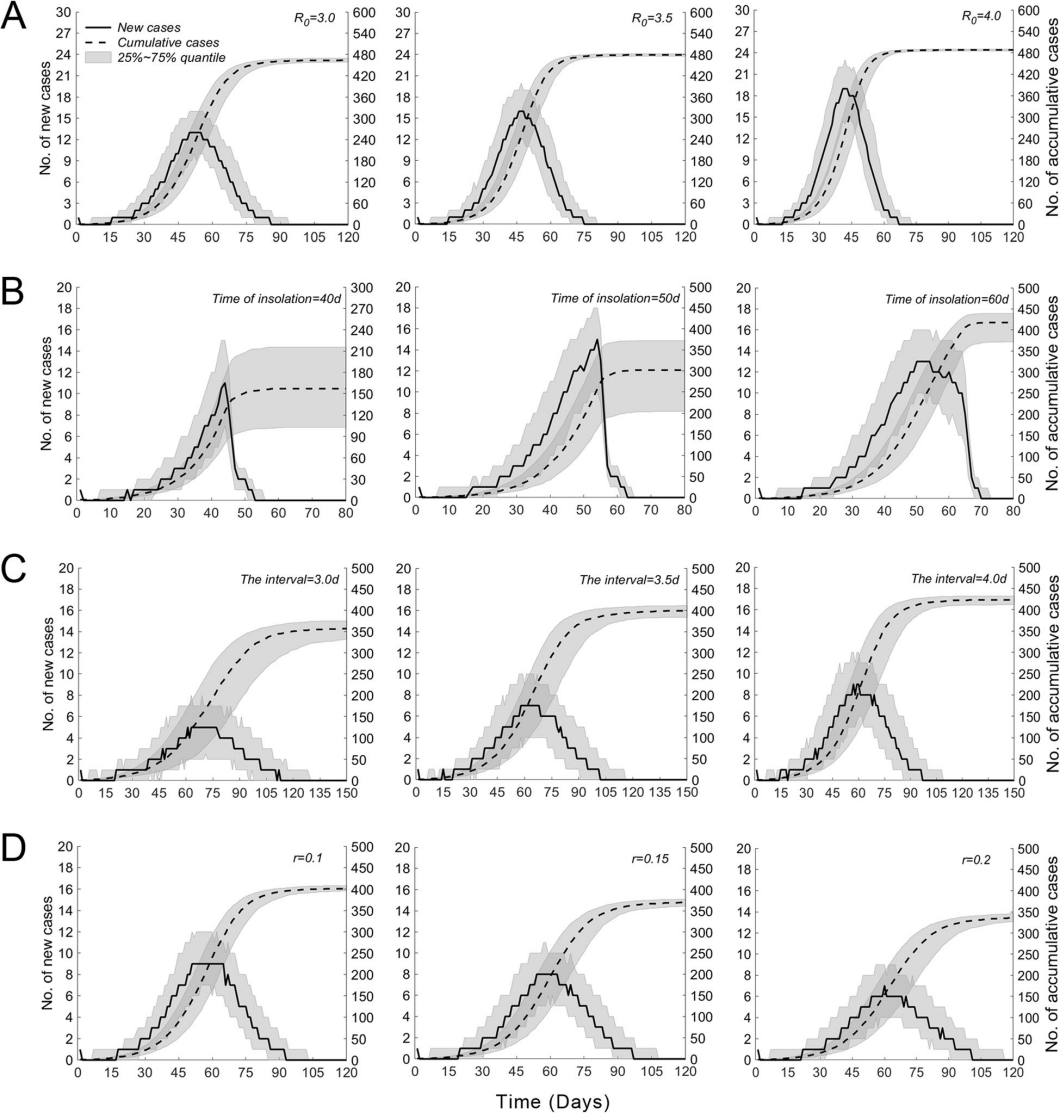
Effect of the four factors on the growth rate of patients and on the cumulative number of patients. Legend: The solid lines represent the growth rate of patients; scales are indicated on the left axis of the coordinate. The dotted lines represent the cumulative number of patients; scales are indicated on the right axis of the coordinate plane. The 25–75% quantiles are indicated by grey shading. **a**, **b**, **c**, and **d** represent the respective effects of *R*_0_, TOI, IOI, and IR on the outbreak, respectively. The above analyses were performed under the following conditions: the total number of individuals exposed in the population was 500; the *R*_0_ for **b**, **c**, and **d** was 3; and the computer simulation was carried out 500 times

***R***_**0**_ As demonstrated in Fig. 3a, when *R*_0_ increased, the attack rate increased correspondingly. The maximum attack rate increased continuously from 0.3 to 0.96. The median attack rate remained close to 0 when *R*_0_ was between 1 and 1.5 but then increased sharply as *R*_0_ increased, reaching a maximum value of 0.93 when *R*_0_ was 3. When the number of patients reaches three or more, the disease is considered an outbreak. We calculated the probability of an outbreak under different *R*_0_ values and found that it rose from close to 0.5 to 0.93. Figure 4a shows that when *R*_0_ was equal to 3, 3.5, and 4, the peak values of the median growth rate (the number of new patients per day) were achieved on the 50th day (13 patients), the 46th day (16 patients), and the 41st day (19 patients), respectively, while the median cumulative number of patients on the 120th day at those *R*_0_ values was 464, 479, and 488 people, respectively. We defined the day that the first patient was detected as the 1st day.

**TOI** Fig. 3b shows that under the condition of *R*_0_ = 3, the probability of an outbreak increased slightly, from 0.85 to 0.9, and consistently stayed near 0.9. When the TOI was on the 10th day, the probability of having more than 10 patients was only 0.2, indicating that the outbreak was well under control. With a delay in the TOI, the probability of having more than 10, 20, 40, or 80 patients increased. When the TOI was later than the 25th day, an outbreak scenario in which more than 80 people were infected began to emerge, indicating that a later TOI leads to infection of more patients and consequently to greater outbreaks. From the 50th day onwards, the attack rate of the epidemic stabilized and remained at a high level. As demonstrated in Fig. 4b, when the TOI was the 40th day and the 50th day, the median growth rate peaked after 4 days (11 patients and 15 patients, respectively) and then dropped rapidly, with corresponding median cumulative numbers of patients of 157 and 300, respectively. When the TOI was on the 60th day, the median cumulative number of patients was 418.

**IOI** Fig. 3c shows that when *R*_0_ was 3, the probability of an outbreak rose from 0.32 to 0.92. The maximum attack rate increased from 0.16 to approximately 0.95, and the 75% quantile, median, and 25% quantile of the attack rate began to increase drastically at days 1.5, 2.3, and 3.8, respectively, with all three approaching 0.9 on the 6th day. This result suggests that when the IOI is below a certain threshold, the attack rate of the disease can be controlled at a low level; however, once the IOI exceeds the threshold, the attack rate will increase very quickly. Figure 4c shows that when the TOI was on day 3, 3.5, and 4, the growth rate of patients peaked on the 63rd day (five patients), 58th day (seven patients), and 57th day (nine patients), respectively, while the median cumulative patient numbers were 357, 400, and 423, respectively.

**IR** Again, at *R*_0_ = 3, the probability of an outbreak showed a continuous reduction from 0.92 to 0.56, as shown in Fig. 3d. At the same time, the maximum attack rate was reduced from 0.96 to 0.2, and the 75% quantile, median, and 25% quantile of the attack rate all dropped from the original value of 0.92. When the IR exceeded 0.5, the median attack rate approached zero. As shown in Fig. 4d, when the IR was 0.1, 0.15, and 0.2, the growth rates for patient numbers peaked at the 56th day (10 patients), the 57th day (eight patients), and the 60th day (seven patients), and the cumulative patient numbers were 402, 370, and 336 patients, respectively.

### Generation period

**Fig. 5:**
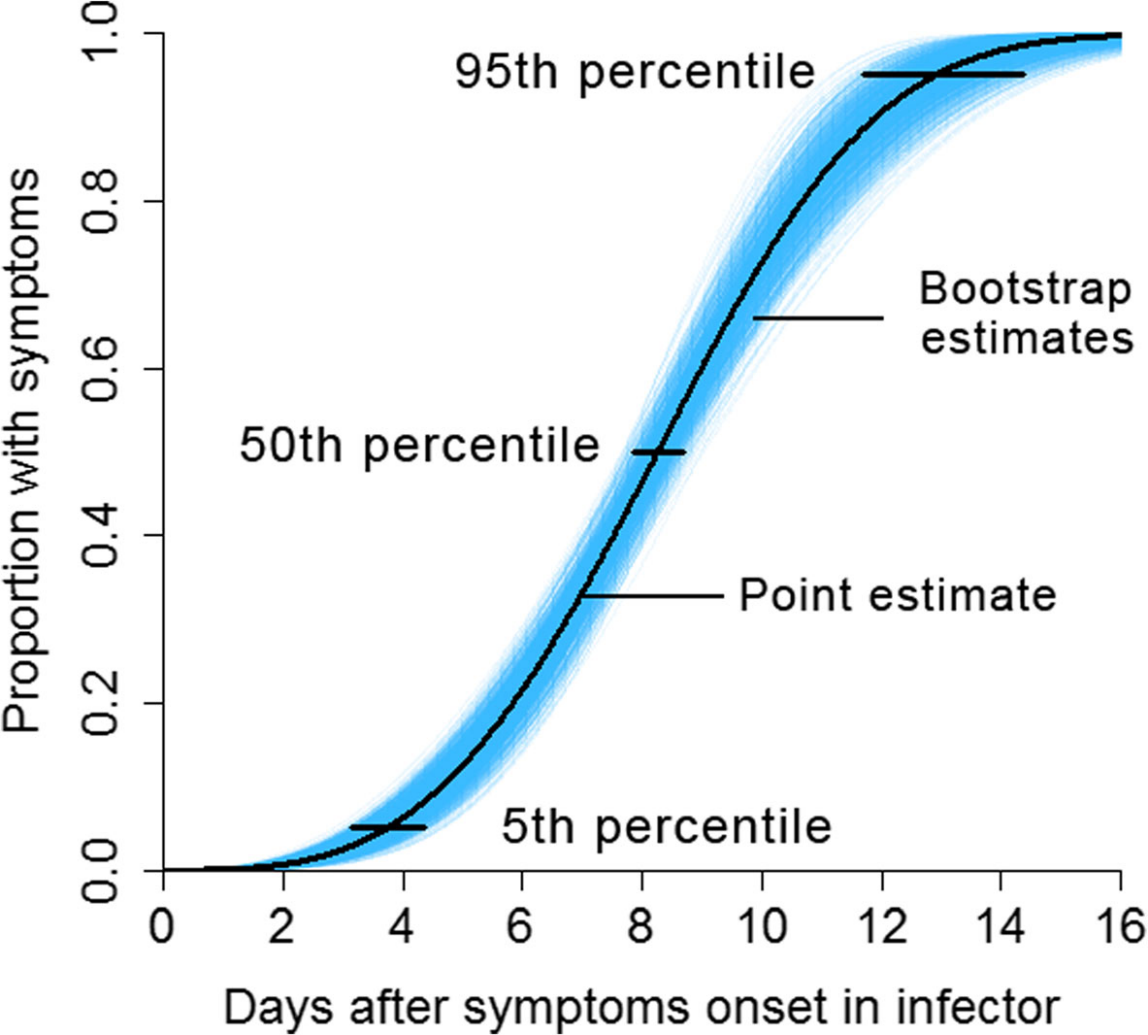
Generation period. Legend: The black curve represents the point estimate for the generation period, and the blue shaded area represents the estimated interval calculated using the bootstrap method. The median was 8.26 days (95% CI: 7.84–8.69 days), with 3.72 days representing the 5% quantile (95% CI: 3.15–4.35 days) and 12.96 days representing the 95% quantile (95% CI: 11.71–14.36 days)

We obtained an average generation period of 8.28 days, with a standard deviation of 2.78 days. Figure 5 shows that the median was estimated to be 8.26 days (95% CI: 7.84–8.69 days).

### Sensitivity analyses

Sensitivity analyses were performed to assess the relationships between the four indexes (*R*_0_, TOI, IOI, IR) and one output (attack rate). We obtained 500 samples from a uniform distribution for each parameter range, and the PRCCs for the four indexes were 0.61, 0.17, 0.45, and − 0.27, respectively. A value greater than 0 indicates a positive correlation, and a value less than 0 indicates a negative correlation. Values near − 1 or + 1 indicate that the parameter has a strong impact on the output, whereas values closer to 0 indicate less effect on the output result.

## Discussion

In our study, we used an idea completely different from the SEIR model. The random collision model has the following specific advantages: 1. The model can more precisely describe the process of epidemic transmission. We used the individual subject as the study unit, and in the programme, each patient’s file contains a record of the times of infection, attack, isolation, and rehabilitation; susceptible persons he might have infected; and whether his range of activities was restricted during the day and night. Not only is this approach conducive to inquiring about the disease development process in each patient, but the transmission chain of the infection can be drawn, enabling in-depth analysis of the transmission path of an infectious disease in a crowd. 2. Randomization is in greater agreement with the transmission characteristics of an infectious disease. Contact between patients and susceptible persons is random rather than continuous. The effective contact rate, incubation period, treatment duration, and immunity of the patients are also in accordance with a random distribution. We randomly sampled from the probability distributions, shown in Table 1, that were obtained from the actual epidemic situation and distributed to each patient. This sampling can ensure the authenticity and scientific integrity of the research. 3. This method can be extended to other infectious diseases and their occurrence scenarios, e.g., tuberculosis outbreaks in schools or the spread of HIV among gay men; we can also set patient activities in programmes, such as in a school where students attend classes during the day and return home at night or board at the school. Furthermore, more complex factors affecting epidemic transmission can be integrated into the programme, providing flexibility and diversity that the SEIR and other traditional models cannot achieve.

Additionally, in the following four paragraphs, we discuss the impact of the four indicators, *R*_0_, TOI, IOI, and IR, on the attack rate in the outbreak.

Overall, *R*_0_ was positively correlated with the attack rate at a PRCC of 0.61, which was the highest absolute value among the four parameters included in the comparison, indicating that *R*_0_ has the strongest influence on the attack rate. Notably, the median attack rate did not continue to increase with an increase in *R*_0_, which differs from the SEIR theory that an epidemic will be triggered once *R*_0_ > 1 [26]; rather, it began to increase dramatically at a certain critical point. The reason for this dramatic increase is that when the value of *R*_0_ is small, even when a source of infection is present within the crowd, a patient’s ability to spread the disease is weak, and he will recover before the disease is transmitted to other susceptible people. As *R*_0_ increases, the speed of disease transmission increases, and the cumulative effect is amplified in a manner that corresponds to the increase in the number of disease generations. This phenomenon reminds us that as long as appropriate preventive measures (such as health education and active circulation of indoor air) are taken to keep *R*_0_ at a low level, serious disease outbreaks can be prevented.

Timely isolation of patients after an outbreak can very effectively control further outbreaks of an epidemic. Our results showed that when the *R*_0_ stays constant, with a delay in the TOI, the probability of a total patient number exceeding 80 people initially remains very low, then rises sharply, and finally reaches a high level and remains there. By contrast, SEIR theory posits that the attack rate will continue to rise as the TOI is delayed. This pattern reveals that when isolation treatment is carried out at the early/beginning stage of an outbreak, the attack rate can be controlled at a lower level; however, with postponement of isolation measures, the total number of patients will increase very quickly, and if isolation is initiated too late, outbreaks of the epidemic become extremely difficult to control. When the TOI occurred before the growth rate peaked, the growth rate displayed a phased trend of an initial increase, followed by a rapid decrease and a final slow decrease. This trend was observed because many people became infected before being isolated; although those who became sick after the TOI were isolated within 1 day of the onset of illness, those patients may have had contact with others and thus could have transmitted the virus. A small number of the individuals infected during this period will become new patients after the incubation period, which averages approximately 5 days.

Timely diagnosis and treatment of patients following early onset of the disease can reduce the number of susceptible people who are infected. The PRCC for the IOI was 0.45, indicating a strong positive correlation. Under a constant *R*_0_, the probability of an outbreak gradually increased as the IOI was extended. The median attack rate remained very low at first, but when the IOI reached a threshold, the attack rate increased rapidly and then slowed. However, according to SEIR theory, no such threshold exists. This trend suggests that we can effectively reduce the risk of an outbreak by taking isolation measures within a certain time frame. The earlier that detection, diagnosis, and isolation are performed, the greater the possibility that the disease attack rate will remain low.

Immunization is an effective approach for preventing infectious diseases. The PRCC between the IR and attack rate was − 0.27, indicating that the higher the IR among the population, the lower the attack rate will be. The results section shows that as the IR increased gradually from 0, the probability of outbreaks decreased steadily. In addition, the median attack rate continued to decrease rapidly until it reached a critical point, after which it remained at an extremely low level; this outcome diverges from the SEIR model, which posits that an epidemic will not occur once the IR increases to *R*_0_ < 1. This outcome occurs because when the IR increases to a certain extent, patients will not be able to continue to infect more susceptible people. This trend indicates that the outbreak of an epidemic can be efficiently restricted if the IR reaches a critical point. However, if it does not reach that critical point, disease prevention will be limited. In general, the relationships between the attack rate and the above four parameters were similar: all displayed a sharp rise in the attack rate after the parameter reached a certain critical value, indicating that the risk of an epidemic outbreak is manageable. Nonetheless, if the measures taken are not effective, the difficulty of controlling the outbreak will increase rapidly.

Although we present some original findings, our study has some limitations. For example, the suitability of the random collision model for diseases that have a more chronic prevalence among the population (such as tuberculosis and AIDS, among others) still requires further discussion, although the value of the SEIR model for these diseases has been confirmed. Because cluster outbreaks have fewer influencing factors and shorter durations, it is relatively easy to establish a random collision model. However, for certain other chronic diseases, modelling requires the consideration of various additional factors, including population migration, age structures, and government interventions. In addition, we calculated only the PRCCs between the attack rate and each parameter in the sensitivity analyses; we did not investigate the compounding effects of multiple parameters acting together on the attack rate, a topic that needs to be addressed in future studies.

## Conclusions

In summary, when the *R*_0_, TOI, and IOI exceed certain thresholds, the disease attack rate increases very quickly, whereas when the immunization rate of the population exceeds the threshold, the disease attack rate declines rapidly. The random collision model is suitable for simulation and epidemiological analysis of outbreaks of respiratory infectious diseases. This model is an extension of the dynamics model of infectious diseases and provides a theoretical basis for individuals to identify opportunities to control an outbreak more precisely and to allocate public health resources in a more rational manner.

## Supplementary information

**Additional file 1.** Algorithm of parameters in **Table 1** and raw data

## Data Availability

The datasets used and/or analysed during the current study available from the corresponding author on reasonable request.

## Abbreviations

ADV 7: Adenovirus type 7
CI: Confidence Interval
IOI: Interval between onset and isolation
IR: Immunization rate
LHS: Latin hypercube sampling
PRCC: Partial rank correlation coefficient
SEIR: Susceptible-Exposed-Infectious-Recovered
TOI: Time of isolation of the patients
