# Supplementary material for: An artificially simulated outbreak of a respiratory infectious disease
Source: BMC Public Health. 2020 Jan 30;20:135. doi: 10.1186/s12889-020-8243-6 (PMC6993344; doi:10.1186/s12889-020-8243-6)
Supplement: Supplementary file 1 — Additional file 1. Algorithm of parameters in Table 1 and raw data [file 12889_2020_8243_MOESM1_ESM.docx]

**Supplemental material for an artificially simulated outbreak of a respiratory infectious disease**

Zuiyuan Guo, Shuang Xu, Libo Tong, Botao Dai, Yuandong Liu, Dan Xiao

Herein, we elaborate on the statistical methods of incubation period, generation time, symptom duration, hospitalization duration, and basic reproductive number provided in table 1 in the methods section of the main text.

An outbreak caused by adenovirus type 7 occurred in a boot camp in China in 2018, which was characterized by a large number of cases, severe symptoms and intrapulmonary infection in many patients. Center for Disease Control and Prevention (CDC) in Northern Theater Command implemented a series of comprehensive prevention and control measures and analysed the following indexes using statistical methods.

Since CDC is responsible for investigating and controlling public health emergencies. The investigation of the adenovirus type 7 epidemic was a public health concern and therefore did not require review by the institutional review board or written informed consent. The responses of the respondents during the investigation were voluntary.

**1. Incubation period**

The incubation period refers to the time period from infection to the initial onset of symptoms. According to published literature, the incubation period *t* of respiratory infections mostly fits a lognormal (log-norm) distribution $\log t\sim N(\mu,\sigma)$[1-3]; the probability density function is shown in formula 1.

$f(t)=\frac{1}{\sqrt{2\pi}\sigma t}e^{-\frac{(\log t-\mu)^{2}}{2\sigma^{2}}}$ $t>0$ (1)

$\mu$ and $\sigma$ represent the mean and standard deviation, respectively. We could only estimate the time ranges of infection and the initial onset of symptoms during the investigation, as the time when the event occurred could not be accurately determined. The range of time *s* of infection was set as $(V_{L},V_{H})$, and the range of time *t* when symptoms appeared was set as $(T_{L},T_{H})$. The probability of infection of and symptoms onset in the *i*^th^ patient could be calculated by formula 2:

$h_{i}(\mu,\sigma;V_{iL},V_{iH},T_{iL},T_{iH})=\int_{T_{iL}}^{T_{iH}} \int_{V_{iL}}^{V_{iH}} g(s)f_{\mu,\sigma}(t-s)dsdt$ $i=1,2,\cdots\cdots,N$ (2)

where $g()$ is the probability of infection and *N* is the total number of patients. We assumed that the infection was uniformly distributed within the time period $s\sim U(V_{L},V_{H})$. During the investigation, we confirmed the transmission chain of the epidemic, including 101 infector-infectee pairs, and determined the time range of infection and symptom onset in the infectee in each pair. We performed a round of random sampling on all pairs and used the maximum likelihood method to establish the maximum likelihood function of the probability of disease onset after infection in *N*=101 patients:

$L(\mu,\sigma)=\prod_{i=1}^{N} h_{i}(\mu,\sigma;V_{iL},V_{iH},T_{iL},T_{iH})$ (3)

We used the logarithm of formula 3 and calculated the partial derivatives of *μ* and *σ* to obtain the estimated values for two parameters during a round of random sampling results to further obtain the cumulative probability curve. We performed 1000 rounds of random sampling and obtained 1000 curves for the cumulative probability distribution function; the medians of the two parameters were $\mu\approx1.64$ and $\sigma\approx0.18$.

Because the transmission relationships of some infector/infectee pairs were not certain, some patients could have had multiple possible infection times. When we performed random sampling on these patients, we only randomly selected one possible infection time. The ranges of the patients’ infection times and symptom onset times are shown in Table s1; the cumulative probability distributions of incubation period is shown in Fig s1A.

**2. Generation time**

Generation time refers to the time between successive onsets of symptoms in an infector-infectee pair with a transmission relationship. Based on 101 infector-infectee pairs, we could find the time range in which the infector first experienced symptoms $(GV_{L},GV_{H})$. According to a previous study, the Weibull distribution (formula 4) reflects the actual distribution for generation time [1]. We therefore used the Weibull distribution to estimate the generation time:

$f(t)=\frac{mt^{m-1}}{\eta^{m}}e^{-(\frac{t}{\eta})^{m}}$ $t>0$ (4)

where $m$ and $\eta$ represent the shape and scale parameters, respectively; the estimation method for the parameters was similar to that for the incubation period. The time when the infector first exhibited symptoms was also within a certain interval, and we assumed that this time fit a uniform distribution. We first established a probability equation for the successive appearance of symptoms in an infector-infectee pair and then performed a round of random sampling in all of the samples. The maximum likelihood method was used to establish the maximum likelihood function, and we finally calculated the parameter estimates for the round of random sampling via the optim() function in R. After 1000 rounds of random sampling, we found that the median parameters were $shape\approx3.48$ and $scale\approx8.19$.

Some infectees might correspond to multiple possible infectors; therefore, the generation time of these pairs had multiple possibilities. During the random sampling, we only randomly selected one possible onset time of infection. The intervals for the onset time for the infector-infectee pairs are shown in Table s2; the cumulative probability distributions of generation time is shown in Fig s1B.

**3. Symptom duration and hospitalization duration**

Symptom duration and hospitalization duration refer to the durations of the clinical symptoms and hospitalization of the patients, respectively. We calculated the symptom durations of 107 patients under treatment and the hospitalization duration of 109 patients who were hospitalized. The ggplot2, ggfortify, survival, and survminer packages were applied in R. The survival curve of the nonparametric Kaplan-Meier method and its confidence interval were plotted via the autoplot() function. Additionally, we also used a parametric method to fit the survival curves for symptom duration and hospitalization duration using log-logistic distribution, Weibull distribution and gamma distribution (using least squares method) and found that the log-logistic function fit the survival curves best. Finally, we used stat_function() to draw the loglogistic function curve. The symptom durations and hospitalization durations of the patients are shown in Table s3; the cumulative probability distributions of symptom duration and hospitalization duration is shown in Fig s1C.


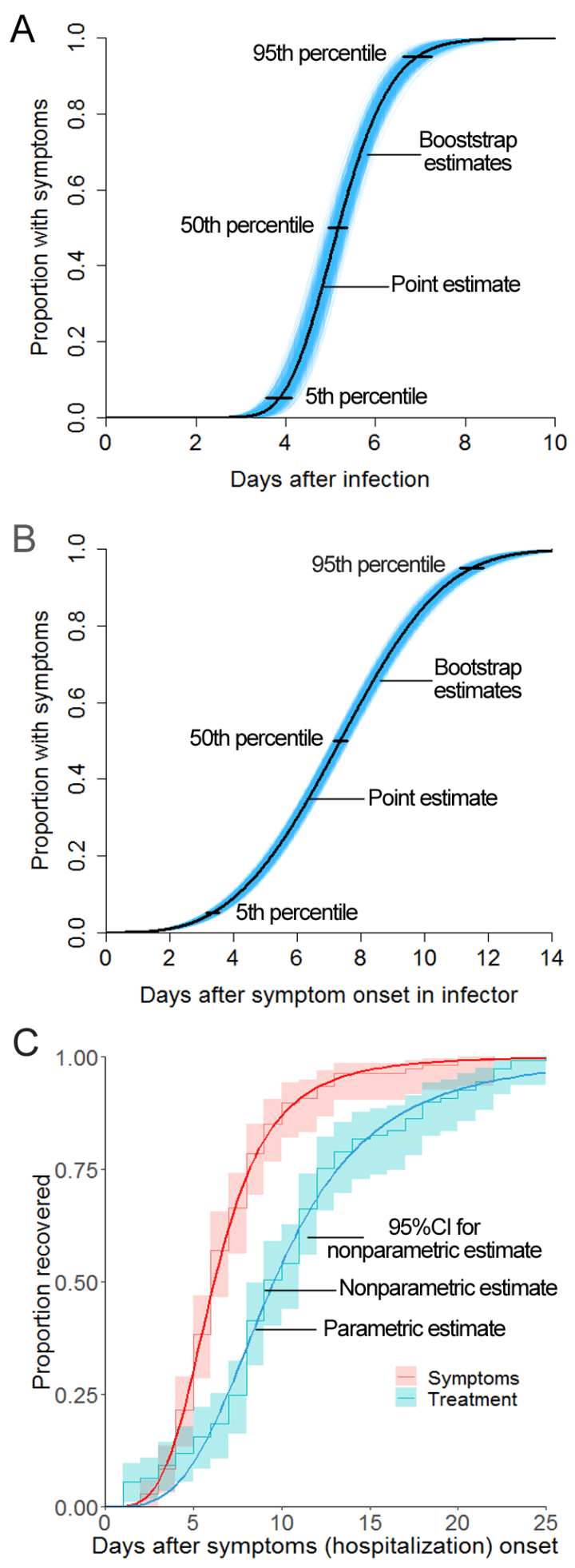


Figure s1. Cumulative probability distributions of the incubation period, generation time, symptom duration and hospitalization duration

Legends: A. Cumulative probability distribution function of the incubation period. The blue region indicates 1000 cumulative probability distribution function curves with lognormal distribution drawn by the bootstrap method, from which the point and interval estimations were obtained. The median incubation period was 5.2 d (95% CI: 5.0 to 5.4 d), with 5% of patients experiencing disease onset within 3.9 d (95% CI: 3.6 to 4.1 d) and 95% of patients experiencing disease onset within 6.9 d (95% CI: 6.6 to 7.2 d). B. Cumulative probability distribution function of the generation time. The blue region represents the interval estimate of Weibull distribution. The median generation time was 7.3 d (95% CI: 7.1 to 7.6 d), with 5% of patients experiencing disease onset within 3.3 d (95% CI: 3.1 to 3.6 d) and 95% of patients experiencing disease onset within 11.5 d (95% CI: 11.1 to 11.8 d). C. Survival curves of symptom duration and hospitalization duration. The non-parametric method estimated that the median symptom duration was 6 d (95% CI: 6 to 7 d), with 25% of patients recovering within 4 d (95% CI: 4 to 5 d) and 75% of patients recovering within 8 d (95% CI: 8 to 9 d), and the median hospitalization duration was 9 d (95% CI: 9 to 11 d), with 25% of patients being discharged within 7 d (95% CI: 6 to 8 d) and 75% of patients being discharged within 12 d (95% CI: 12 to 17 d). The parametric method estimated that the median symptom duration was 6.2 d, with 5% and 95% of patients recovering within 2.9 d and 13.1 d, respectively, and that the median hospitalization duration was 9.6 d, with 5% and 95% of patients being discharged within 4.0 d and 22.6 d, respectively.

**4. Basic reproductive number**

The number of new patients increased exponentially during the early stage of the epidemic with the growth rate $\beta$. Based on this characteristic, we estimated the basic reproductive number. Formula 5 can be used for estimation when the generation time follows a gamma distribution.

$R=(1+\beta\mu\upsilon^{2})^{\frac{1}{\upsilon^{2}}}$ (5)

$\mu$ is the mean generation time and $\upsilon$ is the coefficient of variation. We fitted a gamma distribution of the generation time data, and the mean value and standard deviation were 7.34 and 2.43, respectively. In comparison, the parameters of Weibull distribution are 7.36 and 2.47. We estimated $\beta$ using Formula 6:

$L(\beta|C)\propto\exp(-\beta\sum_{i=0}^{t-1} C(i))(1-\exp(-\beta))^{C(t)-C(0)}$ (6)

where $C(i)$ is the cumulative number of cases at time $i$. We estimated that the exponential growth period was from October 27 to November 12, 2018. The basic reproductive number could be calculated after $\beta$ was acquired using the maximum likelihood method. The supported interval of *R* was calculated based on the interval of $\beta$ according to the maximum likelihood method.

**References**

1. Lessler J, Reich NG, Cumming DAT. Outbreak of 2009 pandemic influenza A (H1N1) at a New York city school. N Engl J Med. 2009;361:2628-36.

2. Lessler J, Reich NG, Brookmeyer R, Perl TM, NelsonKE, Cummings DAT. Incubation periods of acute respiratory viral infections: a systematic review. Lancet Infect Dis. 2009;9:291–300.

3. Reich NG, Lessler J, Cummings DAT, Brookmeyer R. Estimating incubation period distributions with coarse data. Stat Med. 2009;28:2769-84.

4. Becker N. Analysis of Infectious Disease Data. London: Chapman and Hall; 1989.

Table s1. Time intervals of infection and symptom onset in the patients.

| No. | $V_{L}$ | $V_{H}$ | $T_{L}$ | $T_{H}$ | No. | $V_{L}$ | $V_{H}$ | $T_{L}$ | $T_{H}$ |
| --- | --- | --- | --- | --- | --- | --- | --- | --- | --- |
| 1 | Nov.4 | Nov.5 | Nov.10 | Nov.11 | 72 | Nov.10 | Nov.11 | Nov.15 | Nov.16 |
| 2 | Nov.1 | Nov.2 | Nov.4 | Nov.5 |  | Nov.4 | Nov.5 | Nov.15 | Nov.16 |
| 3 | Nov.7 | Nov.8 | Nov.10 | Nov.11 | 73 | Nov.6 | Nov.7 | Nov.10 | Nov.11 |
| 4 | Nov.7 | Nov.8 | Nov.11 | Nov.12 |  | Nov.5 | Nov.6 | Nov.10 | Nov.11 |
| 5 | Nov.7 | Nov.8 | Nov.12 | Nov.13 |  | Nov.4 | Nov.5 | Nov.10 | Nov.11 |
| 6 | Nov.3 | Nov.4 | Nov.9 | Nov.10 | 74 | Nov.4 | Nov.5 | Nov.10 | Nov.11 |
| 7 | Oct.20 | Oct.21 | Oct.29 | Oct.30 |  | Nov.3 | Nov.4 | Nov.10 | Nov.11 |
| 8 | Nov.3 | Nov.4 | Nov.12 | Nov.13 |  | Nov.1 | Nov.2 | Nov.10 | Nov.11 |
| 9 | Nov.3 | Nov.4 | Nov.14 | Nov.15 | 75 | Nov.8 | Nov.9 | Nov.12 | Nov.13 |
| 10 | Nov.7 | Nov.8 | Nov.13 | Nov.14 |  | Nov.7 | Nov.8 | Nov.12 | Nov.13 |
| 11 | Nov.7 | Nov.8 | Nov.15 | Nov.16 |  | Nov.5 | Nov.6 | Nov.12 | Nov.13 |
| 12 | Nov.7 | Nov.8 | Nov.15 | Nov.16 | 76 | Nov.8 | Nov.9 | Nov.14 | Nov.15 |
| 13 | Nov.10 | Nov.11 | Nov.13 | Nov.14 |  | Nov.7 | Nov.8 | Nov.14 | Nov.15 |
| 14 | Nov.10 | Nov.11 | Nov.15 | Nov.16 | 77 | Nov.8 | Nov.9 | Nov.15 | Nov.16 |
| 15 | Nov.3 | Nov.4 | Nov.7 | Nov.8 |  | Nov.7 | Nov.8 | Nov.15 | Nov.16 |
| 16 | Nov.9 | Nov.10 | Nov.13 | Nov.14 | 78 | Nov.9 | Nov.10 | Nov.12 | Nov.13 |
| 17 | Nov.9 | Nov.10 | Nov.13 | Nov.14 |  | Nov.7 | Nov.8 | Nov.12 | Nov.13 |
| 18 | Nov.9 | Nov.10 | Nov.15 | Nov.16 | 79 | Nov.10 | Nov.11 | Nov.13 | Nov.14 |
| 19 | Nov.11 | Nov.12 | Nov.16 | Nov.17 |  | Nov.9 | Nov.10 | Nov.13 | Nov.14 |
| 20 | Nov.11 | Nov.12 | Nov.16 | Nov.17 |  | Nov.7 | Nov.8 | Nov.13 | Nov.14 |
| 21 | Nov.8 | Nov.9 | Nov.11 | Nov.12 | 80 | Nov.10 | Nov.11 | Nov.14 | Nov.15 |
| 22 | Nov.8 | Nov.9 | Nov.14 | Nov.15 |  | Nov.9 | Nov.10 | Nov.14 | Nov.15 |
| 23 | Nov.6 | Nov.7 | Nov.9 | Nov.10 | 81 | Nov.3 | Nov.4 | Nov.10 | Nov.11 |
| 24 | Nov.4 | Nov.5 | Nov.13 | Nov.14 |  | Nov.7 | Nov.8 | Nov.10 | Nov.11 |
| 25 | Nov.4 | Nov.5 | Nov.14 | Nov.15 | 82 | Nov.7 | Nov.8 | Nov.12 | Nov.13 |
| 26 | Nov.7 | Nov.8 | Nov.11 | Nov.12 |  | Nov.3 | Nov.4 | Nov.12 | Nov.13 |
| 27 | Oct.30 | Oct.31 | Nov.9 | Nov.10 | 83 | Nov.10 | Nov.11 | Nov.13 | Nov.14 |
| 28 | Nov.9 | Nov.10 | Nov.12 | Nov.13 |  | Nov.7 | Nov.8 | Nov.13 | Nov.14 |
| 29 | Nov.8 | Nov.9 | Nov.13 | Nov.14 |  | Nov.3 | Nov.4 | Nov.13 | Nov.14 |
| 30 | Nov.9 | Nov.10 | Nov.16 | Nov.17 | 84 | Nov.9 | Nov.10 | Nov.14 | Nov.15 |
| 31 | Nov.9 | Nov.10 | Nov.16 | Nov.17 |  | Nov.8 | Nov.9 | Nov.14 | Nov.15 |
| 32 | Nov.12 | Nov.13 | Nov.15 | Nov.16 |  | Nov.7 | Nov.8 | Nov.14 | Nov.15 |
| 33 | Nov.11 | Nov.12 | Nov.16 | Nov.17 |  | Nov.6 | Nov.7 | Nov.14 | Nov.15 |
| 34 | Nov.3 | Nov.4 | Nov.11 | Nov.12 | 85 | Nov.7 | Nov.8 | Nov.10 | Nov.11 |
| 35 | Nov.3 | Nov.4 | Nov.12 | Nov.13 |  | Nov.5 | Nov.6 | Nov.10 | Nov.11 |
| 36 | Nov.3 | Nov.4 | Nov.12 | Nov.13 | 86 | Nov.12 | Nov.13 | Nov.15 | Nov.16 |
| 37 | Nov.11 | Nov.12 | Nov.14 | Nov.15 |  | Nov.9 | Nov.10 | Nov.15 | Nov.16 |
| 38 | Nov.11 | Nov.12 | Nov.14 | Nov.15 | 87 | Nov.12 | Nov.13 | Nov.15 | Nov.16 |
| 39 | Nov.11 | Nov.12 | Nov.15 | Nov.16 |  | Nov.11 | Nov.12 | Nov.15 | Nov.16 |
| 40 | Nov.4 | Nov.5 | Nov.10 | Nov.11 | 88 | Nov.12 | Nov.13 | Nov.15 | Nov.16 |
| 41 | Nov.4 | Nov.5 | Nov.11 | Nov.12 |  | Nov.11 | Nov.12 | Nov.15 | Nov.16 |
| 42 | Nov.4 | Nov.5 | Nov.12 | Nov.13 | 89 | Nov.11 | Nov.12 | Nov.14 | Nov.15 |
| 43 | Nov.4 | Nov.5 | Nov.12 | Nov.13 |  | Nov.10 | Nov.11 | Nov.14 | Nov.15 |
| 44 | Nov.3 | Nov.4 | Nov.7 | Nov.8 |  | Nov.4 | Nov.5 | Nov.14 | Nov.15 |
| 45 | Nov.3 | Nov.4 | Nov.10 | Nov.11 | 90 | Nov.7 | Nov.8 | Nov.12 | Nov.13 |
| 46 | Nov.11 | Nov.12 | Nov.14 | Nov.15 |  | Nov.3 | Nov.4 | Nov.12 | Nov.13 |
| 47 | Nov.10 | Nov.11 | Nov.13 | Nov.14 | 91 | Nov.10 | Nov.11 | Nov.14 | Nov.15 |
| 48 | Nov.6 | Nov.7 | Nov.9 | Nov.10 |  | Nov.7 | Nov.8 | Nov.14 | Nov.15 |
| 49 | Nov.8 | Nov.9 | Nov.13 | Nov.14 | 92 | Nov.12 | Nov.13 | Nov.15 | Nov.16 |
| 50 | Nov.5 | Nov.6 | Nov.12 | Nov.13 |  | Nov.10 | Nov.11 | Nov.15 | Nov.16 |
| 51 | Nov.5 | Nov.6 | Nov.13 | Nov.14 |  | Nov.7 | Nov.8 | Nov.15 | Nov.16 |
| 52 | Nov.10 | Nov.11 | Nov.15 | Nov.16 | 93 | Nov.12 | Nov.13 | Nov.15 | Nov.16 |
| 53 | Nov.9 | Nov.10 | Nov.15 | Nov.16 |  | Nov.10 | Nov.11 | Nov.15 | Nov.16 |
| 54 | Nov.11 | Nov.12 | Nov.15 | Nov.16 |  | Nov.7 | Nov.8 | Nov.15 | Nov.16 |
| 55 | Nov.10 | Nov.11 | Nov.15 | Nov.16 | 94 | Nov.8 | Nov.9 | Nov.11 | Nov.12 |
| 56 | Nov.8 | Nov.9 | Nov.11 | Nov.12 |  | Nov.6 | Nov.7 | Nov.11 | Nov.12 |
| 57 | Oct.25 | Oct.26 | Nov.1 | Nov.2 | 95 | Nov.9 | Nov.10 | Nov.12 | Nov.13 |
| 58 | Nov.1 | Nov.2 | Nov.11 | Nov.12 |  | Nov.8 | Nov.9 | Nov.12 | Nov.13 |
| 59 | Nov.11 | Nov.12 | Nov.14 | Nov.15 |  | Nov.6 | Nov.7 | Nov.12 | Nov.13 |
| 60 | Nov.11 | Nov.12 | Nov.15 | Nov.16 | 96 | Nov.9 | Nov.10 | Nov.13 | Nov.14 |
| 61 | Oct.29 | Oct.30 | Nov.3 | Nov.4 |  | Nov.8 | Nov.9 | Nov.13 | Nov.14 |
| 62 | Oct.29 | Oct.30 | Nov.4 | Nov.5 |  | Nov.6 | Nov.7 | Nov.13 | Nov.14 |
| 63 | Oct.30 | Oct.30 | Nov.4 | Nov.5 | 97 | Nov.10 | Nov.11 | Nov.15 | Nov.16 |
| 64 | Oct.30 | Oct.30 | Nov.5 | Nov.6 |  | Nov.8 | Nov.9 | Nov.15 | Nov.16 |
| 65 | Oct.30 | Oct.30 | Nov.5 | Nov.6 | 98 | Nov.12 | Nov.13 | Nov.16 | Nov.17 |
| 66 | Oct.30 | Oct.30 | Nov.6 | Nov.7 |  | Nov.10 | Nov.11 | Nov.16 | Nov.17 |
| 67 | Nov.6 | Nov.7 | Nov.16 | Nov.17 | 99 | Nov.11 | Nov.12 | Nov.15 | Nov.16 |
| 68 | Nov.9 | Nov.10 | Nov.13 | Nov.14 |  | Nov.8 | Nov.9 | Nov.15 | Nov.16 |
| 69 | Nov.5 | Nov.6 | Nov.8 | Nov.9 | 100 | Nov.11 | Nov.12 | Nov.16 | Nov.17 |
| 70 | Nov.5 | Nov.6 | Nov.9 | Nov.10 |  | Nov.8 | Nov.9 | Nov.16 | Nov.17 |
| 71 | Nov.4 | Nov.5 | Nov.13 | Nov.14 | 101 | Nov.6 | Nov.7 | Nov.14 | Nov.15 |
|  | Nov.10 | Nov.11 | Nov.13 | Nov.14 |  | Nov.5 | Nov.6 | Nov.14 | Nov.15 |

Note: the time intervals of infection for patients No. 1-70 were confirmed, and the time intervals of infection for patients No. 71-101 were not confirmed (multiple possibilities).

Table s2. The time intervals of sequential disease onset for infectors and infectees.

| No. | $U_{L}$ | $U_{H}$ | $T_{L}$ | $T_{H}$ | No. | $U_{L}$ | $U_{H}$ | $T_{L}$ | $T_{H}$ |
| --- | --- | --- | --- | --- | --- | --- | --- | --- | --- |
| 1 | Nov.2 | Nov.3 | Nov.10 | Nov.11 | 72 | Nov.9 | Nov.10 | Nov.15 | Nov.16 |
| 2 | Oct.30 | Oct.31 | Nov.4 | Nov.5 |  | Nov.2 | Nov.3 | Nov.15 | Nov.16 |
| 3 | Nov.5 | Nov.6 | Nov.10 | Nov.11 | 73 | Nov.6 | Nov.7 | Nov.10 | Nov.11 |
| 4 | Nov.6 | Nov.7 | Nov.11 | Nov.12 |  | Nov.3 | Nov.4 | Nov.10 | Nov.11 |
| 5 | Nov.5 | Nov.6 | Nov.12 | Nov.13 |  | Nov.2 | Nov.3 | Nov.10 | Nov.11 |
| 6 | Nov.1 | Nov.2 | Nov.9 | Nov.10 | 74 | Nov.2 | Nov.3 | Nov.10 | Nov.11 |
| 7 | Nov.18 | Nov.19 | Nov.29 | Nov.30 |  | Nov.1 | Nov.2 | Nov.10 | Nov.11 |
| 8 | Nov.2 | Nov.3 | Nov.12 | Nov.13 |  | Oct.30 | Oct.31 | Nov.10 | Nov.11 |
| 9 | Nov.1 | Nov.2 | Nov.14 | Nov.15 | 75 | Nov.7 | Nov.8 | Nov.12 | Nov.13 |
| 10 | Nov.5 | Nov.6 | Nov.13 | Nov.14 |  | Nov.6 | Nov.7 | Nov.12 | Nov.13 |
| 11 | Nov.6 | Nov.7 | Nov.15 | Nov.16 |  | Nov.4 | Nov.5 | Nov.12 | Nov.13 |
| 12 | Nov.5 | Nov.6 | Nov.15 | Nov.16 | 76 | Nov.6 | Nov.7 | Nov.14 | Nov.15 |
| 13 | Nov.9 | Nov.10 | Nov.13 | Nov.14 |  | Nov.5 | Nov.6 | Nov.14 | Nov.15 |
| 14 | Nov.8 | Nov.9 | Nov.15 | Nov.16 | 77 | Nov.6 | Nov.7 | Nov.15 | Nov.16 |
| 15 | Nov.2 | Nov.3 | Nov.7 | Nov.8 |  | Nov.5 | Nov.6 | Nov.15 | Nov.16 |
| 16 | Nov.8 | Nov.9 | Nov.13 | Nov.14 | 78 | Nov.8 | Nov.9 | Nov.12 | Nov.13 |
| 17 | Nov.8 | Nov.9 | Nov.13 | Nov.14 |  | Nov.6 | Nov.7 | Nov.12 | Nov.13 |
| 18 | Nov.7 | Nov.8 | Nov.15 | Nov.16 | 79 | Nov.8 | Nov.9 | Nov.13 | Nov.14 |
| 19 | Nov.9 | Nov.10 | Nov.16 | Nov.17 |  | Nov.7 | Nov.8 | Nov.13 | Nov.14 |
| 20 | Nov.9 | Nov.10 | Nov.16 | Nov.17 |  | Nov.5 | Nov.6 | Nov.13 | Nov.14 |
| 21 | Nov.6 | Nov.7 | Nov.11 | Nov.12 | 80 | Nov.8 | Nov.9 | Nov.14 | Nov.15 |
| 22 | Nov.7 | Nov.8 | Nov.14 | Nov.15 |  | Nov.7 | Nov.8 | Nov.14 | Nov.15 |
| 23 | Nov.4 | Nov.5 | Nov.9 | Nov.10 | 81 | Nov.1 | Nov.2 | Nov.10 | Nov.11 |
| 24 | Nov.2 | Nov.3 | Nov.13 | Nov.14 |  | Nov.5 | Nov.6 | Nov.10 | Nov.11 |
| 25 | Nov.2 | Nov.3 | Nov.14 | Nov.15 | 82 | Nov.5 | Nov.6 | Nov.12 | Nov.13 |
| 26 | Nov.6 | Nov.7 | Nov.11 | Nov.12 |  | Nov.1 | Nov.2 | Nov.12 | Nov.13 |
| 27 | Oct.29 | Oct.30 | Nov.10 | Nov.11 | 83 | Nov.8 | Nov.9 | Nov.13 | Nov.14 |
| 28 | Nov.7 | Nov.8 | Nov.12 | Nov.13 |  | Nov.5 | Nov.6 | Nov.13 | Nov.14 |
| 29 | Nov.6 | Nov.7 | Nov.13 | Nov.14 |  | Nov.1 | Nov.2 | Nov.13 | Nov.14 |
| 30 | Nov.7 | Nov.8 | Nov.16 | Nov.17 | 84 | Nov.7 | Nov.8 | Nov.14 | Nov.15 |
| 31 | Nov.7 | Nov.8 | Nov.16 | Nov.17 |  | Nov.6 | Nov.7 | Nov.14 | Nov.15 |
| 32 | Nov.10 | Nov.11 | Nov.15 | Nov.16 |  | Nov.5 | Nov.6 | Nov.14 | Nov.15 |
| 33 | Nov.10 | Nov.11 | Nov.16 | Nov.17 |  | Nov.4 | Nov.5 | Nov.14 | Nov.15 |
| 34 | Nov.1 | Nov.2 | Nov.11 | Nov.12 | 85 | Nov.6 | Nov.7 | Nov.10 | Nov.11 |
| 35 | Nov.1 | Nov.2 | Nov.12 | Nov.13 |  | Nov.4 | Nov.5 | Nov.10 | Nov.11 |
| 36 | Nov.1 | Nov.2 | Nov.12 | Nov.13 | 86 | Nov.10 | Nov.11 | Nov.15 | Nov.16 |
| 37 | Nov.9 | Nov.10 | Nov.14 | Nov.15 |  | Nov.7 | Nov.8 | Nov.15 | Nov.16 |
| 38 | Nov.9 | Nov.10 | Nov.14 | Nov.15 | 87 | Nov.10 | Nov.11 | Nov.15 | Nov.16 |
| 39 | Nov.9 | Nov.10 | Nov.15 | Nov.16 |  | Nov.9 | Nov.10 | Nov.15 | Nov.16 |
| 40 | Nov.2 | Nov.3 | Nov.10 | Nov.11 | 88 | Nov.11 | Nov.12 | Nov.15 | Nov.16 |
| 41 | Nov.2 | Nov.3 | Nov.11 | Nov.12 |  | Nov.10 | Nov.11 | Nov.15 | Nov.16 |
| 42 | Nov.2 | Nov.3 | Nov.12 | Nov.13 | 89 | Nov.9 | Nov.10 | Nov.14 | Nov.15 |
| 43 | Nov.2 | Nov.3 | Nov.12 | Nov.13 |  | Nov.8 | Nov.9 | Nov.14 | Nov.15 |
| 44 | Nov.1 | Nov.2 | Nov.7 | Nov.8 |  | Nov.2 | Nov.3 | Nov.14 | Nov.15 |
| 45 | Nov.1 | Nov.2 | Nov.10 | Nov.11 | 90 | Nov.6 | Nov.7 | Nov.12 | Nov.13 |
| 46 | Nov.9 | Nov.10 | Nov.14 | Nov.15 |  | Nov.2 | Nov.3 | Nov.12 | Nov.13 |
| 47 | Nov.9 | Nov.10 | Nov.13 | Nov.14 | 91 | Nov.8 | Nov.9 | Nov.14 | Nov.15 |
| 48 | Nov.5 | Nov.6 | Nov.9 | Nov.10 |  | Nov.5 | Nov.6 | Nov.14 | Nov.15 |
| 49 | Nov.6 | Nov.7 | Nov.13 | Nov.14 | 92 | Nov.11 | Nov.12 | Nov.15 | Nov.16 |
| 50 | Nov.3 | Nov.4 | Nov.12 | Nov.13 |  | Nov.9 | Nov.10 | Nov.15 | Nov.16 |
| 51 | Nov.4 | Nov.5 | Nov.13 | Nov.14 |  | Nov.6 | Nov.7 | Nov.15 | Nov.16 |
| 52 | Nov.9 | Nov.10 | Nov.15 | Nov.16 | 93 | Nov.10 | Nov.11 | Nov.15 | Nov.16 |
| 53 | Nov.7 | Nov.8 | Nov.15 | Nov.16 |  | Nov.8 | Nov.9 | Nov.15 | Nov.16 |
| 54 | Nov.9 | Nov.10 | Nov.15 | Nov.16 |  | Nov.5 | Nov.6 | Nov.15 | Nov.16 |
| 55 | Nov.8 | Nov.9 | Nov.15 | Nov.16 | 94 | Nov.7 | Nov.8 | Nov.11 | Nov.12 |
| 56 | Nov.7 | Nov.8 | Nov.11 | Nov.12 |  | Nov.5 | Nov.6 | Nov.11 | Nov.12 |
| 57 | Oct.29 | Oct.30 | Nov.1 | Nov.2 | 95 | Nov.7 | Nov.8 | Nov.12 | Nov.13 |
| 58 | Oct.30 | Oct.31 | Nov.11 | Nov.12 |  | Nov.6 | Nov.7 | Nov.12 | Nov.13 |
| 59 | Nov.10 | Nov.11 | Nov.14 | Nov.15 |  | Nov.4 | Nov.5 | Nov.12 | Nov.13 |
| 60 | Nov.9 | Nov.10 | Nov.15 | Nov.16 | 96 | Nov.7 | Nov.8 | Nov.13 | Nov.14 |
| 61 | Oct.29 | Oct.30 | Nov.3 | Nov.4 |  | Nov.6 | Nov.7 | Nov.13 | Nov.14 |
| 62 | Oct.29 | Oct.30 | Nov.4 | Nov.5 |  | Nov.4 | Nov.5 | Nov.13 | Nov.14 |
| 63 | Oct.29 | Oct.30 | Nov.4 | Nov.5 | 97 | Nov.8 | Nov.9 | Nov.15 | Nov.16 |
| 64 | Oct.29 | Oct.30 | Nov.5 | Nov.6 |  | Nov.6 | Nov.7 | Nov.15 | Nov.16 |
| 65 | Oct.29 | Oct.30 | Nov.5 | Nov.6 | 98 | Nov.11 | Nov.12 | Nov.16 | Nov.17 |
| 66 | Oct.30 | Oct.31 | Nov.6 | Nov.7 |  | Nov.9 | Nov.10 | Nov.16 | Nov.17 |
| 67 | Nov.4 | Nov.5 | Nov.16 | Nov.17 | 99 | Nov.9 | Nov.10 | Nov.15 | Nov.16 |
| 68 | Nov.7 | Nov.8 | Nov.13 | Nov.14 |  | Nov.6 | Nov.7 | Nov.15 | Nov.16 |
| 69 | Nov.4 | Nov.5 | Nov.8 | Nov.9 | 100 | Nov.10 | Nov.11 | Nov.16 | Nov.17 |
| 70 | Nov.3 | Nov.4 | Nov.9 | Nov.10 |  | Nov.7 | Nov.8 | Nov.16 | Nov.17 |
| 71 | Nov.2 | Nov.3 | Nov.13 | Nov.14 | 101 | Nov.4 | Nov.5 | Nov.14 | Nov.15 |
|  | Nov.8 | Nov.9 | Nov.13 | Nov.14 |  | Nov.3 | Nov.4 | Nov.14 | Nov.15 |

Note: the time intervals of disease onset for infectors No. 1-70 were confirmed, and the time intervals of disease onset for infectors No. 71-101 were not confirmed (multiple possibilities).

Table s3. Symptom duration and treatment duration for the patients (in days).

| No. | Symptom period | Treatment period | No. | Symptom period | Treatment period |
| --- | --- | --- | --- | --- | --- |
| 1 | 2 | 1 | 55 | 6 | 9 |
| 2 | 2 | 1 | 56 | 6 | 10 |
| 3 | 2 | 1 | 57 | 6 | 10 |
| 4 | 3 | 1 | 58 | 6 | 10 |
| 5 | 3 | 1 | 59 | 6 | 10 |
| 6 | 3 | 1 | 60 | 6 | 11 |
| 7 | 3 | 2 | 61 | 6 | 11 |
| 8 | 3 | 3 | 62 | 7 | 11 |
| 9 | 3 | 3 | 63 | 7 | 11 |
| 10 | 4 | 3 | 64 | 7 | 11 |
| 11 | 4 | 4 | 65 | 7 | 11 |
| 12 | 4 | 4 | 66 | 7 | 11 |
| 13 | 4 | 4 | 67 | 7 | 11 |
| 14 | 4 | 5 | 68 | 7 | 11 |
| 15 | 4 | 5 | 69 | 7 | 11 |
| 16 | 4 | 5 | 70 | 7 | 11 |
| 17 | 4 | 5 | 71 | 7 | 11 |
| 18 | 4 | 6 | 72 | 8 | 11 |
| 19 | 4 | 6 | 73 | 8 | 12 |
| 20 | 4 | 6 | 74 | 8 | 12 |
| 21 | 4 | 7 | 75 | 8 | 12 |
| 22 | 4 | 7 | 76 | 8 | 12 |
| 23 | 4 | 7 | 77 | 8 | 12 |
| 24 | 5 | 7 | 78 | 8 | 12 |
| 25 | 5 | 7 | 79 | 8 | 12 |
| 26 | 5 | 7 | 80 | 8 | 12 |
| 27 | 5 | 7 | 81 | 8 | 12 |
| 28 | 5 | 8 | 82 | 8 | 12 |
| 29 | 5 | 8 | 83 | 8 | 13 |
| 30 | 5 | 8 | 84 | 8 | 13 |
| 31 | 5 | 8 | 85 | 9 | 13 |
| 32 | 5 | 8 | 86 | 9 | 13 |
| 33 | 5 | 8 | 87 | 9 | 14 |
| 34 | 5 | 8 | 88 | 9 | 14 |
| 35 | 5 | 8 | 89 | 9 | 14 |
| 36 | 5 | 8 | 90 | 9 | 15 |
| 37 | 5 | 8 | 91 | 9 | 16 |
| 38 | 5 | 8 | 92 | 10 | 17 |
| 39 | 5 | 8 | 93 | 10 | 17 |
| 40 | 5 | 8 | 94 | 10 | 17 |
| 41 | 5 | 8 | 95 | 10 | 18 |
| 42 | 6 | 8 | 96 | 10 | 18 |
| 43 | 6 | 8 | 97 | 11 | 18 |
| 44 | 6 | 8 | 98 | 12 | 18 |
| 45 | 6 | 8 | 99 | 12 | 19 |
| 46 | 6 | 9 | 100 | 12 | 20 |
| 47 | 6 | 9 | 101 | 13 | 20 |
| 48 | 6 | 9 | 102 | 13 | 21 |
| 49 | 6 | 9 | 103 | 13 | 21 |
| 50 | 6 | 9 | 104 | 17 | 22 |
| 51 | 6 | 9 | 105 | 18 | 22 |
| 52 | 6 | 9 | 106 | 20 | 22 |
| 53 | 6 | 9 | 107 | 22 | 23 |
| 54 | 6 | 9 | 108 |  | 23 |
|  |  |  | 109 |  | 25 |

Table s4. Dormitory transmission chain and number of dormitories in the transmission chain.

| $v$ | $I_{0}$ | $I_{1}$ | $I_{2}$ | $I_{3}$ |
| --- | --- | --- | --- | --- |
| 27 | 1 | 0 | 0 | 0 |
| 10 | 1 | 1 | 0 | 0 |
| 6 | 1 | 2 | 0 | 0 |
| 3 | 1 | 3 | 0 | 0 |
| 1 | 1 | 4 | 0 | 0 |
| 6 | 2 | 2 | 0 | 0 |
| 1 | 2 | 6 | 0 | 0 |
| 1 | 3 | 1 | 0 | 0 |
| 1 | 3 | 2 | 0 | 0 |
| 1 | 4 | 1 | 0 | 0 |
| 2 | 1 | 1 | 2 | 0 |
| 1 | 1 | 1 | 3 | 0 |
| 1 | 1 | 1 | 5 | 0 |
| 1 | 1 | 2 | 1 | 0 |
| 1 | 1 | 2 | 2 | 0 |
| 1 | 1 | 1 | 1 | 2 |
| 1 | 1 | 1 | 2 | 2 |

Note: *v* represents the number of dormitories, and *I_i_* represents the number of patients at the *i^th^* generation of the transmission chain.
